# Irritable bowel syndrome in adults: Prevalence and risk factors

**DOI:** 10.1016/j.amsu.2022.104408

**Published:** 2022-08-19

**Authors:** Muhammad Hamayl Zeeshan, Naga Praneeth Vakkalagadda, Gummadi Sai Sree, Krishna kishore Anne, Sunita devi, Om Parkash, Shaikh Basiq Ul Fawwad, Syed Muhammad Waqar Haider, Hassan Mumtaz, Mohammad Hasan

**Affiliations:** a Dow University of Health Sciences, Pakistan; b Intern, Government General Hospital, Guntur Medical College, Guntur, India; c National Pirogov Memorial Medical University, Ukraine; d Liaquat University of Medical and Health Sciences, Jamshoro, Pakistan; e Jinnah Sindh Medical University, Pakistan; f Maroof International Hospital Islamabad, Public Health Scholar, Health Services Academy Islamabad, Pakistan; g Jinnah Postgraduate Medical Centre (JPMC), Pakistan

**Keywords:** Irritable bowel syndrome, Gastrointestinal disorders, Prevalence, Risk factors

## Abstract

**Introduction:**

The prevalence of irritable bowel syndrome (IBS) ranges from 7 to 18% over the world. We aimed to assess the prevalence and risk factors of irritable bowel syndrome in adults.

**Methodology:**

We conducted a cross-sectional study of IBS prevalence and risk factors from March to May 2022 at KRL Hospital Islamabad. 300 people were given Pre-validated Performa's. Our research adheres to the principles outlined in the Helsinki Declaration. The PSS was used to measures how much stress a person has felt in the past month.

The higher the score, the more stressed the person appears to be. A variety of mental health disorders can be evaluated using this method. Data on dietary and lifestyle factors associated with IBS for the last 12 months was also collected from the participants.

**Results:**

The majority of patients, 70%, were classed as Grade 1 and 146 (48.66%) reported abdominal pain associated with defecation. 162 (54%) individuals reported high levels of tea consumed, 81 (27%) consumed coffee and 57 (19%) reported carbonated drinks consumed. 139 individuals reported having Vigorous-Intensity activity, out of which 69 (49.64%) spend 60 min of vigorous activity in a day.

**Conclusion:**

Screening patients for IBS on a regular basis is critical, especially in the younger demographic. If a patient experiences any symptoms of IBS, they should contact their doctor immediately. Consider the care of patients with chronic gastrointestinal complaints, particularly in women and those at greater risk of developing the illness.

## Highlights

• ***Perceived Stress Scale (PSS)*** measures how much stress a person has felt in the past month.
• The Chalder Fatigue Scale (CFS) is used to measure fatigue.
• We recommend the younger population to be screened for IBS on a regular basis.
• Persistent gastrointestinal symptoms, especially women and those at high risk of the condition, it is critical to take this into account.

## 1 Introduction

Abdominal pain or discomfort linked with defecation or a change in bowel habits are common symptoms of irritable bowel syndrome (IBS) [1]. There are four subtypes of IBS based on their prevailing bowel patterns: diarrhoea-predominant, constipation predominance (IBS C), both diarrhoea and constipation (IBS M), or when the stool pattern cannot be defined under any of the aforementioned three patterns (IBS–U) [2].

For doctors, diagnosing IBS can be difficult because symptoms can change over time [3,4], and the lack of a gold standard makes it a significant difficulty. The Rome criteria were formulated in 2006 and are updated on a regular basis [2] as a result of this. According to Rome IV, the most recent revision of these criteria was issued in 2016. Abdominal distension is accompanied by symptoms of constipation, diarrhoea, or both. Signs and symptoms should have been present for at least three months prior to the diagnosis.

Suicidal thoughts and depression are more common in IBS patients [5], and there is a considerable decline in health-related quality of life, which has an adverse effect on an individual's ability to contribute to society [6]. Women and teenagers are more likely to suffer from this condition than men, according to epidemiological studies [7,8].

It's also important to diagnose the condition immediately from the patient's perspective. Long term IBS can be exasperating for the patient. Costs such as appointments with the doctor, multiple laboratory tests and investigations and prescription of therapeutic drugs can cause a significant economic burden on the patient. Many people with IBS have also complained that they had to take a sick leave from work because of their condition [8].

Although researchers have attempted to identify patterns in incidence and risk factors for this illness, many of them are still not clearly defined. Our aim for conducting this research was because we want to improve the knowledge gap on the prevalence and risk factors that can lead up to irritable bowel syndrome in adults.

## 2 Methods

We conducted a cross-sectional study of IBS prevalence and risk factors from March to May 2022 at KRL Hospital Islamabad. There were 300 people in the study, thus we used the WHO calculator to estimate the sample size. The non-probability sampling method was used in this study.

All individuals with IBS who visited the gastrointestinal outpatient department were included in this study. Under-18s, those who refused to participate and reported a list of red flag symptoms and organic disorders that could be mistaken for IBS were excluded from the study. History of recurrent rectal bleeding, weight loss, and a family history of colorectal cancer are red flag symptoms. Inflammatory bowel disease, colon cancer, celiac disease, and lactose intolerance are examples of organic disorders.

Pre-validated Performa's were presented to patients, and then an 8-page questionnaire via Google Forms was used to collect data. In accordance with the STROCSS 2021 recommendations [9], we conducted this study. As an added bonus, a detailed STROCSS 2021 check list may be found in the supplemental materials. UIN researchregistry7993 [10] identifies our study in Research Registry. Our research adheres to the principles outlined in the Helsinki Declaration. Ethical approval was given by KRL Hospital, Islamabad.

### 2.1 Sociodemographic profile

Sociodemographic details such as age, sex, residence, marital status, and monthly income were asked in the questionnaire.

### 2.2 Perceived Stress Scale (PSS)

PSS is an instrument that measures how much stress a person has felt in the past month. A variety of mental health disorders can be evaluated using this method. Originally, the PSS was made up of 14 parts. A score of 0 (never) to 4 (always) is given to each item (very often). In order to get a total score of between 0 and 40, the negative and positive items' scores are added together. The higher the score, the more stressed the person appears to be [11].

### 2.3 Diet and lifestyle

Data on dietary and lifestyle factors associated with IBS for the last 12 months was also collected from the participants. Diet included regular or irregular mealtimes, frequency of grain consumption, and consumption of milk products, fruits, sweet and salty food. Lifestyle factors included exercise (daily/once a week/once a moth/never), smoking, caffeine and alcohol intake.

### 2.4 Fatigue

The Chalder Fatigue Scale (CFS) is used to measure fatigue [12]. This consists of an 11-item questionnaire consisting of questions about physical changes, decreased ability to think and difficulty in memory. The 11 items are divided into 2 subcategories; 7 for physical fatigue and 4 for mental fatigue. Each score is measured from 0 (better) till 3 (much worse). The total score ranges around 0–33 in which 0–21 indicates physical fatigue and 0–12 indicates mental fatigue.

## 3 Results

**Fig. 1:**
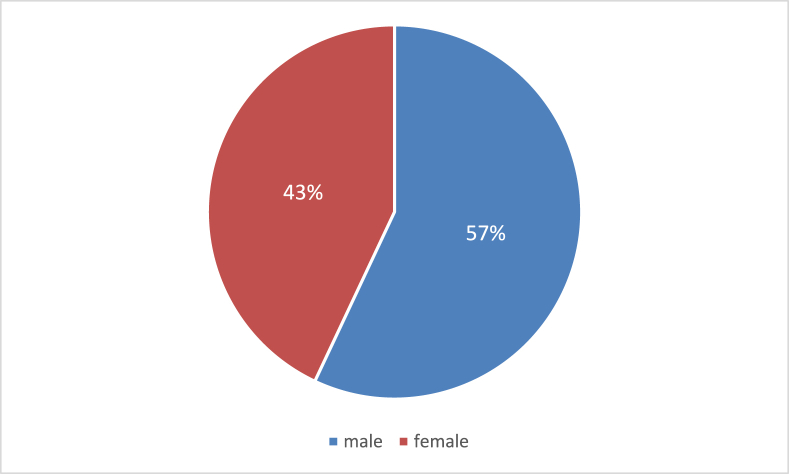
Showing the gender.

A total of 300 patients with irritable bowel syndrome were included in the study. Out of the 300, 171 patients were male (57%) and 129 were female patients (43%), as shown in Fig. 1.

**Fig. 2:**
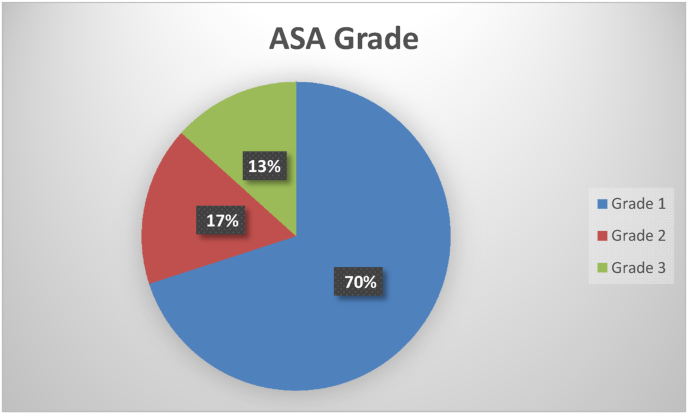
Asa grade.

ASA grading was also used to categorize patients. ASA grade 1 patients are those who are in good physical condition, have a body mass index (BMI) under 30, and do not smoke. Asperger's syndrome (AS) grade 2 patients have a modest systemic illness. Grade 3 patients were those who had a serious illness, but not one that was life-threatening (such as poorly managed hypertension or obesity). Patients in Grade 4 have a serious systemic condition that poses a persistent threat to their lives (for example, unstable angina). ASA Grade 4 patients are those who are predicted to die without surgery. The majority of patients, 210 (70%), were classed as Grade 1 once the final data were analyzed. Fig. 2 shows that 50 patients (17%) were grouped into ASA grade 2 and the rest of the patients (13%) were categorized into ASA grades 3, 4 and 5.

**Fig. 3:**
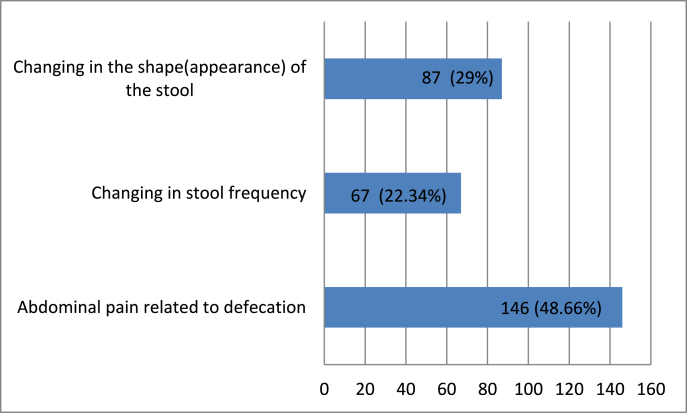
Did you experience any of the following symptoms (at least once a week for the past 3months)?.

Patients were also asked about associating symptoms. Among the 300 patients, 146 (48.66%) reported abdominal pain associated with defecation, 87 (29%) patients reported change in the appearance (shape) of the stool and 67 (22.34%) patients reported change in frequency of their stools, as shown in Fig. 3.

**Fig. 4:**
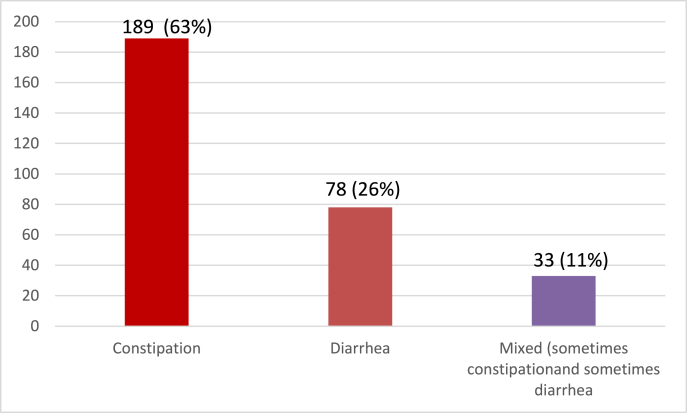
Symptoms experienced the most during the past six months.

Results for the most commonly experienced symptoms were also collected. 189 patients (63%) reported having constipation while 78 patients (26%) reported having diarrhoea for the last 6 months. 33 patients (11%) reported having both (mixed symptoms), as shown in Fig. 4.

**Fig. 5:**
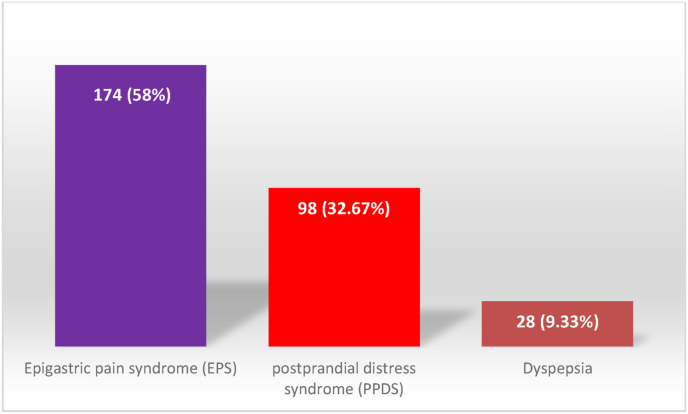
History of patients.

Patients had a history of associating disease as well. 174 patients (58%) reported having Epigastric pain syndrome (EPS), 98 patients (32.67%) had postprandial distress syndrome (PPDS) whereas 28 (9.33%) had dyspepsia, as shown in Fig. 5.

**Fig. 6:**
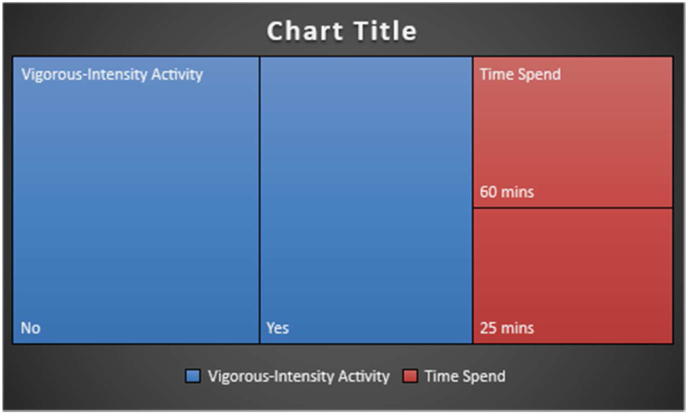
Moderate-intensity activity.

Daily activity of IBS patients was also recorded. 139 individuals reported having Vigorous-Intensity activity, out of which 69 (49.64%) spend 60 min of vigorous activity in a day whereas 70 (50.36%) individuals spent only 25 min per day, as shown in Fig. 6.

**Fig. 7:**
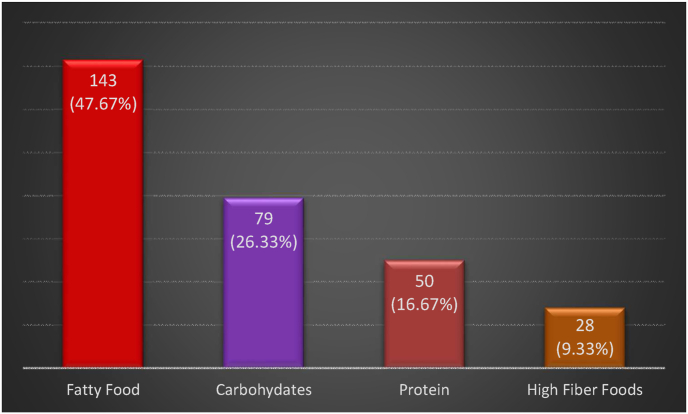
Food pattern predominating in daily diet.

Regarding diet, 143 individuals (47.67%) reported having high Fatty Food diet, while 79 (26.33%) had high carbohydrates diet, 50 individuals (16.67%) had high Protein intake and 28 patients (9.33%) had a high fiber diet, as shown in Fig. 7.

**Fig. 8:**
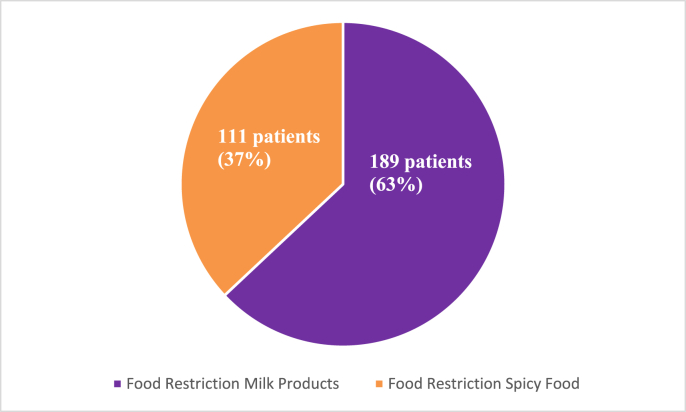
Food restrictions.

A lot of patients had restrictions of food products. 189 patients (63%) reported having food restrictions of milk products whereas 111 individuals (37%) had spicy food restrictions, as shown in Fig. 8.

**Fig. 9:**
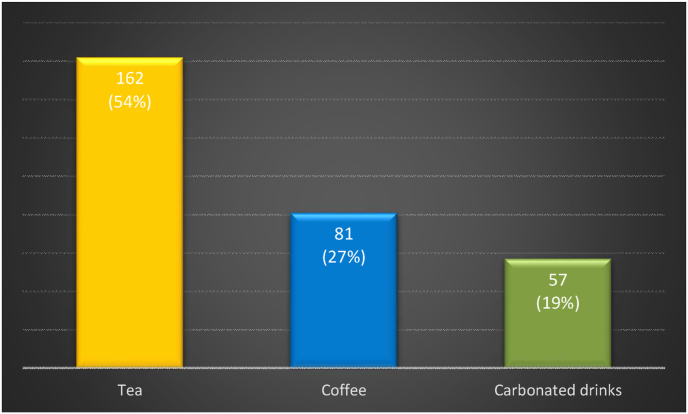
Stimulants consumed.

Data regarding intake of stimulants was also taken. 162 (54%) individuals reported having high levels of tea consumed, 81 (27%) consumed coffee and 57 (19%) reported consuming carbonated drinks, as shown in Fig. 9.

## 4 Discussion

IBS affects between 9% and 23% of the population [13]. Diarrhoea, constipation, or both are common symptoms of IBS, as is abdominal pain. IBS may be diagnosed if there are no other serious symptoms and no other organic illness [14].

Numerous variables contribute to the prevalence of IBS. These characteristics can be based on gender, age, or socioeconomic status. Overall, females are more likely to have IBS, but our results reveal that males have a higher prevalence than females [[15], [16]].

Additionally, it's possible that psychological variables play a role in worsening IBS symptoms. According to the hypothesis put forth by the brain-gut axis [17], psychological influences on gastrointestinal motility are linked. Several pathways can be involved, such as neurological, neuro-immune, or neuroendocrine. When the sympathetic outflow tract is activated, patients with a high level of CNS stimulation are more likely to suffer from IBS symptoms (such as anxiety) [18] because of this. IBS symptoms are more likely to develop in children and adolescents because of this [19].

IBS is characterised by severe abdominal pain [20]. Patients are distressed, and their quality of life suffers as a result [21]. Patients have also expressed frustration that these symptoms have hampered their ability to perform their daily tasks, including going to work and participating in social activities. We found that several of our patients had stomach pain during defecating, as well as a change in the appearance and frequency of their faeces [22]. Abdominal discomfort severity was found to differ between men and women in a study by Rurgo et al. [23]. Males experienced greater pain intensity than females.

As a general rule, IBS-D is more prevalent than IBS-C in the general population [24]. Although, our investigation announced a different outcome, there are certain studies such as a study by Dong et al. [25] in Northern China showing that IBS-C was more commonly seen. IBS subtypes may range from region to region or ethnicity to culture, and this may be seen in this study. Different subtypes of IBS exist. It is possible to classify IBS sufferers into three categories: IBS-C (constipation), IBS-D (diarrhoea), and IBS-M (mixed) [26]. Research has showed that IBS-C and IBS-D are more frequent. Our findings are in line with this study.

Irritable bowel syndrome (IBS) is frequently brought on by foods. More than half of patients report that their symptoms are linked to their food intake, according to a recent study [27]. It's been shown in numerous research that certain foods can cause symptoms [28], such as spicy or fried food, milk and coffee in particular. Our findings are also in line with recent studies, which found that patients with IBS limited their intake of milk and spicy foods.

IBS sufferers might use herbal remedies in addition to diet and lifestyle changes to alleviate any associated side effects. If you have IBS, you may want to consider pursuing this option, even if there have been only a few clinical trials [29].

Patients with IBS may benefit from treatment that includes dietary and lifestyle changes as well as medication. FODMAP (fermentable oligo, di and monosaccharides and polyols), more fibre, less beans, onions, and other foods that may cause gas may help alleviate symptoms of the condition. Lactose-intolerant patients are put on a special diet that excludes all dairy products. Improving the symptoms of IBS can be accomplished with the use of a wide variety of pharmaceuticals. Antidepressants such as tricyclic antidepressants, SSRIs, loperamide, and laxatives [30] are among them.

## 5 Conclusion

It is important to regularly screen patients for IBS, particularly the younger generation. Patients should be made aware that whenever they show any symptom for IBS then they should inform their clinician.

It is important to consider the care of patients with chronic gastrointestinal symptoms, especially in women and those at high risk of developing the condition.

## Ethical approval

Ethical approval was obtained from the ethical committee of KRL Hospital Islamabad, Ref ERC: KRL–HI–ERC/Dec/29826.

## Funding

No Funding Received.

## Author contribution

The main concept was determined by Muhammad Hamayl Zeeshan, Collection of data is done by Hassan Mumtaz, Data is analyzed and interpreted by Naga Praneeth Vakkalagadda, Krishna Kishore Anne, Gummadi Sai Sree, Writing of the manuscript is done by Sunita Devi, Om Prakash, Muhammad Hasan, Manuscript editing is done by Shaikh Basiq Ul Fawwad, Syed Muhammad Waqar Haider.

## Registration of research studies

Name of the registry: Research Registry.

Unique Identifying number or registration ID: researchregistry7993.

Hyperlink to your specific registration (must be publicly accessible and will be checked https://www.researchregistry.com/browse-the-registry#home/registrationdetails/62a2ebe8a4d1e3001fc668e4/

## Guarantor

Hassan Mumtaz & Muhammad Hamayl Zeeshan.

## Provenance and peer review

Not commissioned, externally peer-reviewed.

## Declaration of competing interest

No conflict of interest declared.
